# A case of paraplegia that developed 6 years after thoracic endovascular aortic repair for blunt traumatic aortic injury

**DOI:** 10.1002/ams2.307

**Published:** 2017-09-14

**Authors:** Hiroyuki Takahashi, Tomohisa Shoko, Fumino Taketazu, Keiichi Kuriyama, Kazuhide Yoshikawa, Yoshizumi Deguchi

**Affiliations:** ^1^ Department of Emergency and Critical Care Medicine Tokyo Women's Medical University Medical Center East Tokyo Japan

**Keywords:** Accidents, paraplegia, spinal cord, thoracic injuries, traffic

## Abstract

**Case:**

Thoracic endovascular aortic repair (TEVAR) is becoming the standard therapy for blunt thoracic aortic injury (BTAI). However, the long‐term outcomes of TEVAR for BTAI remain unclear. A 36‐year‐old man was admitted to our emergency department with dyspnea. He had been involved in a serious traffic accident 6 years earlier, requiring TEVAR for BTAI.

**Outcome:**

Acute heart failure and pneumonia were diagnosed on this admission. His respiratory condition improved, but paraplegia developed 10 h after hospitalization. Magnetic resonance imaging showed an intraspinal longitudinal area of signal hyperintensity, and spinal cord infarction was diagnosed.

**Conclusion:**

Although the causal relationship between the TEVAR and spinal cord infarction remains unclear, paraplegia as a long‐term complication after TEVAR does not appear to have been reported previously, and so represents a potentially important complication.

## Introduction

Thoracic endovascular aortic repair (TEVAR) for blunt thoracic aortic injury (BTAI) offers lower rates of mortality and complications than open surgical repair.^1^, ^2^, ^3^ However, the long‐term outcomes of TEVAR for BTAI remain unclear. We report a case in which paraplegia developed 6 years after TEVAR for BTAI.

## Case

A 36‐year‐old man was admitted to our emergency department with a 5‐day history of increasing dyspnea. He had been involved in a traffic accident 6 years earlier, during which he had suffered a BTAI (pseudoaneurysm; Society of Vascular Surgery^4^ grade III, Japanese Association for the Surgery of Trauma Classification^5^ IIIa) requiring treatment by TEVAR. The proximal side of the stent graft was in the subclavian artery, and the distal side was in the descending aorta at the Th6 level. Systemic arterial hypertension was also apparent. He had been transferred to a rehabilitation hospital, but had stopped going to the hospital after discharge because he was busy with work.

On admission, vital signs were as follows: Glasgow Coma Scale score, 15 (E4V5M6); blood pressure, 219/109 mmHg; heart rate, 112 b.p.m.; respiratory rate, 38 breaths/min; peripheral oxygen saturation, 78% in room air, 98% with O_2_ at 10 L/min; and axillary body temperature, 37.4°C. Although his blood pressure was high on admission, he did not know his regular blood pressure.

Because respiratory difficulty was severe, non‐invasive ventilation was provided soon after admission. The first non‐invasive ventilation setting was continuous positive airway pressure, and the fraction of inspired oxygen was 50%. In this setting, arterial blood gas examination showed: pH, 7.469; pCO_2_, 29.4 mmHg; pO_2_, 88.4 mmHg; and ${\text{HCO}}_{3}^{-}$, 21.1 mmol/L. The main findings from blood examinations were: hemoglobin, 10.8 g/dL; albumin, 2.8 g/dL; C‐reactive protein, 23.43 mg/dL; creatinine, 0.77 mg/dL; glucose, 115 mg/dL; international normalized ratio of prothrombin time, 1.29; fibrin/fibrinogen degradation products, 8.6 μg/mL; and N‐terminal pro‐brain natriuretic peptide, 3,858 pg/mL.

**Figure 1:**
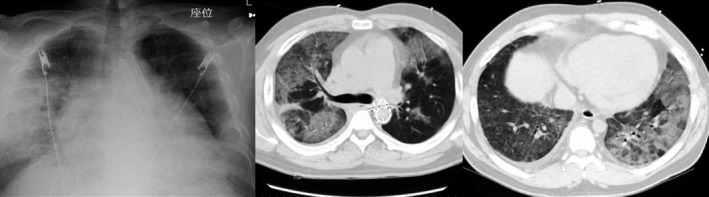
X‐ray and computed tomography of the chest of a 36‐year‐old man 6 years after thoracic endovascular aortic repair for blunt traumatic aortic injury. Chest X‐ray shows cardiac dilatation and an infiltrative shadow in the right middle lung field. Computed tomography of the chest shows infiltrative shadows in lung fields on both sides.

Chest X‐ray and computed tomography (CT) showed cardiac dilatation and an infiltrative shadow in bilateral lung fields (Fig. 1). Electrocardiography showed sinus tachycardia, and no ischemic changes were detected. Echocardiography showed hyposystole (ejection fraction, 47.2%) and left ventricular wall hyperplasia.

Based on these results, acute heart failure (clinical scenario 1^6^) and pneumonia were diagnosed. Acute heart failure was mainly treated using vasodilators, and pneumonia was treated with antibiotics.

Eight hours after hospitalization, systolic blood pressure had gradually decreased to approximately 150 mmHg, and mean arterial pressure had decreased to approximately 100 mmHg. No unexpected sudden drop in blood pressure occurred, the monitor electrocardiogram showed sinus rhythm and no arrhythmias such as atrial fibrillation were evident. Respiratory condition improved, but paraplegia developed 10 h after hospitalization. Both lower limbs showed complete paralysis, and sensation below the Th10 level was completely lost.

**Figure 2:**
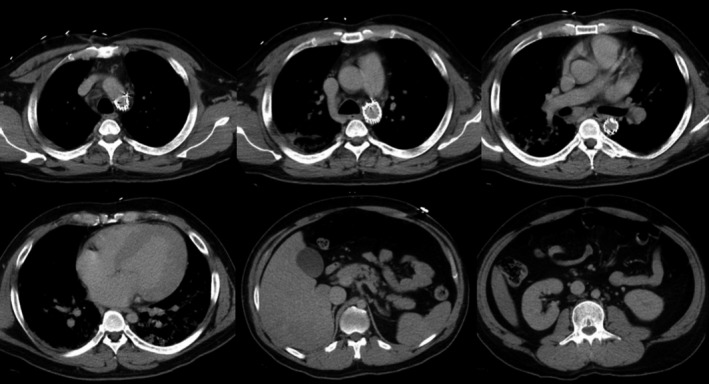
Emergency contrast‐enhanced computed tomography of a 36‐year‐old man taken 6 years after thoracic endovascular aortic repair for blunt traumatic aortic injury. Emergency contrast‐enhanced computed tomography does not show aortic dissection. The aortic stent graft and aorta beyond the stent graft display no blood flow degradation, but the aorta beyond the stent graft is narrowed. Maximum diameter of the thoracic aorta is 15 mm, and that of the abdominal aorta is 16 mm.

Emergency contrast‐enhanced CT did not reveal any aortic dissection. Both within the aortic stent graft and in the aorta beyond the stent graft, no blood flow degradation was evident, but the aorta beyond the stent graft was narrowed (Fig. 2). Neither the artery of Adamkiewicz nor collateral artery supplies to the spinal cord were identified on this emergency contrast‐enhanced CT.

**Figure 3:**
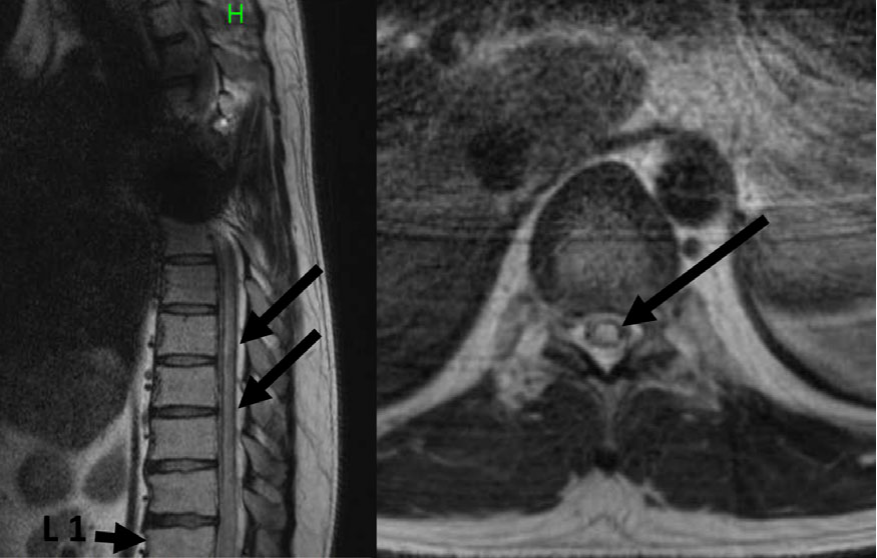
Emergency magnetic resonance imaging of the thoracolumbar junction of a 36‐year‐old man, taken 6 years after thoracic endovascular aortic repair for blunt traumatic aortic injury. Magnetic resonance imaging of the thoracolumbar junction shows an intraspinal, longitudinal area of signal hyperintensity from the Th7 to Th11 level, although evaluation above the Th7 level was made difficult due to the artifact created by the stent graft. The “H” means the “head” direction of the figure and the arrows show an intraspinal, longitudinal area of signal hyperintensity.

Magnetic resonance imaging of the thoracolumbar junction showed an intraspinal, longitudinal area of signal hyperintensity from the Th7 to Th11 level (Fig. 3). Spinal cord infarction was diagnosed.

Although blood pressure remained high, the relative drop in blood flow might have contributed to the infarction, so further reductions in pressure were prevented.

Thereafter, the respiratory condition continued to improve. However, no improvement of paraplegia was achieved, and the patient was transferred to a rehabilitation hospital on day 53 after hospitalization.

## Discussion

We encountered a case of paraplegia that occurred 6 years after TEVAR for BTAI. Although the causal relationship between the TEVAR and spinal cord infarction remains unclear, paraplegia as a long‐term complication after TEVAR does not appear to have been reported previously, and so represents a potentially important complication.

Paraplegia after thoracoabdominal or thoracic aortic repair is considered to represent myelopathy due to spinal cord ischemia,^7^ a known serious complication. Rates of paraplegia after treatment for BTAI have been reported as 2.2–5.4% for patients who undergo operative repair, and 0–1.4% for those who undergo TEVAR.^1^, ^2^, ^3^ All comparative reports have suggested that paraplegia is less frequent with TEVAR than with open surgical repair. Some recent reports have described long‐term findings of TEVAR for BTAI.^8^, ^9^, ^10^ In those reports, the median age at TEVAR was 39–43 years, and the 5‐year survival rate was >80%. No cases of paraplegia were described. Complications such as endoleak or thrombosis have been described, but were small in number. Long‐term results following TEVAR for BTAI thus seem good overall.

Martin *et al*.^10^ reported that seven of 60 patients who underwent TEVAR showed systemic arterial hypertension shortly after TEVAR, and none of those seven patients had a history of hypertension before the procedure. As our case also showed hypertension after TEVAR, the underlying mechanism might involve the maintenance of blood flow downstream of the stent‐graft, or a reaction against the increased afterload resulting from stent‐graft placement.

One of the main postoperative risk factors for delayed neurological deficit after thoracic and thoracoabdominal aortic aneurysm repair is lower mean arterial pressure below 60 mmHg.^7^ In our case, when the spinal cord infarction developed, mean arterial pressure was approximately 100 mmHg, which was not considered low. However, spinal cord infarction was attributed to a relative decrease in blood flow resulting from multiple effects such as blood pressure control for acute heart failure, hypoxia, anemia, and coagulopathy due to inflammation, on the basis of the hypertensive compensatory mechanisms for relative ischemia by BTAI itself or TEVAR.

Current guidelines suggest that TEVAR be used preferentially over open surgical repair, regardless of age.^11^ Similarly, guideline findings should be tempered by the current lack of knowledge regarding the natural history of endovascular repair and optimal follow‐up strategies. Therefore, for patients undergoing TEVAR for BTAI, long‐term follow‐up is warranted to cope with any unexpected abnormalities.

## Conclusion

We encountered a case of paraplegia occurring 6 years after TEVAR for BTAI. Thoracic endovascular aortic repair for BTAI is an effective treatment that is gaining popularity, however, long‐term follow‐up is warranted.

## Disclosure

Written informed consent was obtained from the patient for publication of this case report.

Conflict of interest: Authors declare no Conflict of Interest for this article.
